# Using exosomes for universal vaccines

**DOI:** 10.1128/iai.00129-25

**Published:** 2025-07-09

**Authors:** Alfred R. Babich

**Affiliations:** 1 Unaffiliated Researcher, Buffalo, New York, USA; University of California Davis, Davis, California, USA

**Keywords:** exosomes, mucosal vaccines, vaccines, influenza vaccines

## LETTER

The article recently published by Emerson et al. (1) represents a major step forward in vaccination methods. This is particularly true in view of the recent findings published by Gurriaran-Rodriguez et al. (2), which reveal the identity of the exosome-binding peptide sequence. The genetic sequence for this peptide sequence may be inserted into the genetic sequence of any protein. That sequence may then be introduced into cells such as mesenchymal cells, monocytes, and possibly macrophages activated with gamma interferon. They would then produce exosomes containing that protein, which could be a bacterial or viral antigen. The significance of this is vast in view of Emerson’s findings that antigen-containing vesicles can induce mucosal immunity.

One possible group of antigenic proteins that could be directed into the exosomes would be the adhesins and pilins found in bacterial cell walls. These proteins are essential for bacterial colonization and invasion, making them a perfect target for vaccines capable of inducing mucosal immunity. Furthermore, there is evidence that they contain highly conserved epitopes (3, 4), which would explain the success of this current round of experiments. This means that broad-spectrum intranasal antibacterial vaccines might be produced to induce mucosal immunity. These vaccines could stop both infection and colonization for both gram-positive and gram-negative bacteria, including troublesome microbes such as *Escherichia coli* and *Staphylococcus aureus*.

Also very significant were the findings that the vesicles could induce T cell immunity. This is particularly true as highly conserved areas of the cell-binding proteins of cold and influenza viruses are inaccessible to antibodies (5). If these epitopes from cold or influenza viruses could be introduced into exosomes, they might be used in universal cold and influenza vaccines functioning through T cell immunity.

At this point, Emerson’s group should probably stay with their current approach, as their research is already at an advanced stage. Also, there is no way to know if artificially induced exosomes would have the same immunogenic effect as naturally produced extracellular vesicles, as it is not known if the exosomes would have to contain particular cytokines for them to have the desired immunogenic effect. Their next steps should probably be finding how to produce their vaccine at an industrial scale and proceeding to human trials. Nonetheless, the findings by Gurriaran-Rodriguez et al. (2) should lay out the pathway for the next generation of vaccines. With diarrheal disease being a major cause of childhood death in developing countries and antibiotic resistance being an increasing problem everywhere, we would be most grossly remiss if we did not aggressively pursue this important new avenue of research.
